# Distinctive physical properties of DNA shared by RNA polymerase II gene promoters and 5′-flanking regions of tRNA genes

**DOI:** 10.1093/jb/mvad111

**Published:** 2023-12-15

**Authors:** Kohei Uemura, Takashi Ohyama

**Affiliations:** Major in Integrative Bioscience and Biomedical Engineering, Graduate School of Science and Engineering, Waseda University, 2-2 Wakamatsu-cho, Shinjuku-ku, Tokyo 162-8480, Japan; Major in Integrative Bioscience and Biomedical Engineering, Graduate School of Science and Engineering, Waseda University, 2-2 Wakamatsu-cho, Shinjuku-ku, Tokyo 162-8480, Japan; Department of Biology, Faculty of Education and Integrated Arts and Sciences, Waseda University, 2-2 Wakamatsu-cho, Shinjuku-ku, Tokyo 162-8480, Japan

**Keywords:** Core promoter, lncRNA, miRNA, physical properties of DNA, protein-coding gene, tRNA

## Abstract

Numerous noncoding (nc)RNAs have been identified. Similar to the transcription of protein-coding (mRNA) genes, long noncoding (lnc)RNA genes and most of micro (mi)RNA genes are transcribed by RNA polymerase II (Pol II). In the transcription of mRNA genes, core promoters play an indispensable role; they support the assembly of the preinitiation complex (PIC). However, the structural and/or physical properties of the core promoters of lncRNA and miRNA genes remain largely unexplored, in contrast with those of mRNA genes. Using the core promoters of human genes, we analyzed the repertoire and population ratios of residing core promoter elements (CPEs) and calculated the following five DNA physical properties (DPPs): duplex DNA free energy, base stacking energy, protein-induced deformability, rigidity and stabilizing energy of Z-DNA. Here, we show that their CPE and DPP profiles are similar to those of mRNA gene promoters. Importantly, the core promoters of these three classes of genes have two highly distinctive sites in their DPP profiles around the TSS and position −27. Similar characteristics in DPPs are also found in the 5′-flanking regions of tRNA genes, indicating their common essential roles in transcription initiation over the kingdom of RNA polymerases.

## Graphical Abstract

**Graphical Abstract:**
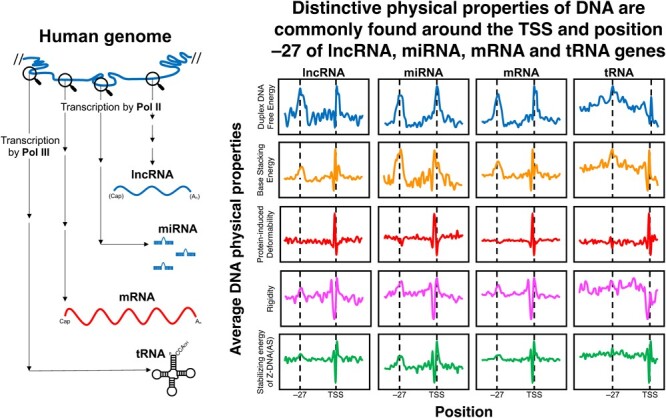

## Abbreviations

CAGE: cap analysis of gene expression
CPE: core promoter element
DHS: DNase I hypersensitive site
DPE: downstream promoter element
DPP: DNA physical property
Inr: initiator
lncRNA: long noncoding RNA
miRNA: micro RNA
ncRNA: noncoding RNA
PIC: pre-initiation complex
Pol II: RNA polymerase II
Pol III: RNA polymerase III
TBP: TATA-binding protein
TSS: transcription start site
Z-DNA (AS): Z-DNA (anti-syn)

A promoter is a DNA region where RNA polymerase binds and initiates transcription. Among the promoters of various classes of genes, those of mRNA genes have been extensively studied, clarifying the various sequence features of core promoters *(**1**,**2**)*. Core promoters are the region that supports the assembly of the preinitiation complex (PIC) *(**1**,**3**)*, comprising the general transcription factors TFIIA, TFIIB, TFIID, TFIIE, TFIIF, TFIIH and RNA polymerase II (Pol II) in its minimal form. In some PICs, the promoters harbor core promoter element (CPEs) of 6–10 bp with defined sequences, including a TATA box, initiator (Inr) and downstream promoter element (DPE) *(**2**)*. These CPEs have long been believed necessary to initiate faithful transcription.

In transcription of lncRNA genes and most of miRNA genes, Pol II is also used. Numerous ncRNAs have been identified by recent progress in methodology, including deep-sequencing of transcriptomes *(**4**,**5**)*. LncRNAs, defined as > 200 nucleotides ncRNAs, have been proposed to have diverse functions, including X-chromosome inactivation and imprinting, although the biological functions of the vast majority remain enigmatic *(**6*–*8**)*. MiRNAs are ~ 22 nucleotides ncRNAs that mediate gene silencing via binding to target mRNAs or inhibition of translation initiation *(**9*–*12**)*, and may influence all developmental processes and diseases in humans *(**10**,**11**)*. Although the promoters of lncRNA and miRNA genes and the regulation of their expression have also been extensively studied *(**13*–*18**)*, the knowledge is not yet comparable to that for protein-coding genes. Even the structural and physical properties of the core promoters of lncRNA and miRNA genes remain poorly explored, in stark contrast to those of the core promoters of mRNA genes.

Many studies have suggested that the structural and physical properties of the promoter DNA, rather than the sequences of the CPEs, may play an essential role in the transcription of mRNA genes *(**19*–*24**)*. These studies have identified two specific regions with peculiar structural and physical properties within the core promoters of mRNA genes: around the TSS and the region corresponding to the TATA box. Furthermore, using a mammalian transient expression system, Fukue *et al*. showed that the physical properties themselves were indispensable to drive transcription *(**20**)*. Based on these studies, the core promoters of ncRNA genes that are transcribed by Pol II, such as lncRNA genes and miRNA genes, may also have peculiar structural and/or physical properties around the TSS and TATA-corresponding regions to execute faithful transcription. However, no comprehensive or comparative studies of the structural and/or physical properties among the core promoters of mRNA, lncRNA and miRNA genes have been performed. Using these promoters and 5′-flanking regions of tRNA genes, we analyzed the repertoire and population ratios of the component CPEs, calculated five DPPs: duplex DNA free energy, base stacking energy, protein-induced deformability, rigidity and stabilizing energy of Z-DNA (AS) (AS: anti-syn), and found an interesting common feature in their DPP profiles.

## Methods

### Genome sequences and data sets for TSSs

The whole human genome sequences (hg19 and hg38) were downloaded from the UCSC Genome Browser. The TSSs of human lncRNAs and mRNAs were obtained from the FANTOM CAT robust cutoff *(**25**)* and evaluated with TIEScore, and the TSS with the highest TIEScore was chosen as the representative TSS of a gene. The promoters of lncRNA genes were divided into 16 groups on the basis of the DNase I hypersensitive site (DHS) type annotation and the gene orientation annotation defined by Hon *et al*. *(**25**)*. The promoters of human miRNA genes were obtained from the integrated expression atlas of miRNAs reported by de Rie *et al*. *(**16**)*. Human tRNA sequences were from hg38. High-confidence tRNA TSSs with single-nucleotide resolution were from Yan *et al*. *(**26**)*, and the TSS with the highest TPM (transcripts per million) of a given gene was used. The positions of the 5′-end and the discriminator of a mature tRNA were obtained from GtRNAdb *(**27**)*.

### CPE analysis

Sequences of CPEs were obtained from Haberle and Stark *(**2**)*. Deviations of ±5 bp from their canonical positions were allowed. Promoters lacking any CPEs were treated as core-less promoters *(**20**)*. Using R (ver. 4.2.2), 3000 sequences of mRNA gene promoters were randomly collected.

### Data set of DPPs

Five DPPs were used in the acquisition of DPP profiles. These were duplex DNA free energy *(**28**)*, base stacking energy *(**29**)*, protein-induced deformability [a sequence with a larger deformability value (the values were defined by Olson *et al*.) is easily deformed by proteins, while that with a smaller value is less susceptible to structural changes by proteins] *(**30**)*, rigidity *(**31**)*, and stabilizing energy of Z-DNA (AS) *(**32**)*.

**Figure 1:**
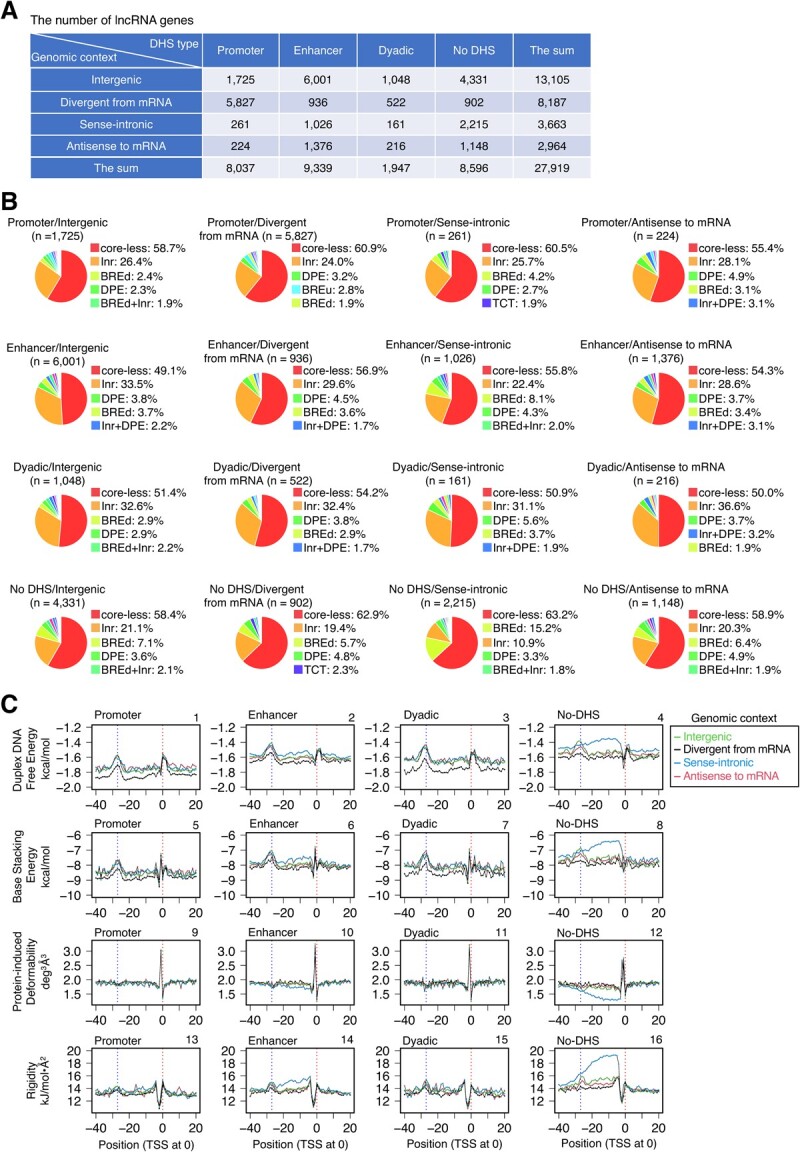
Continues

**Fig. 1:**
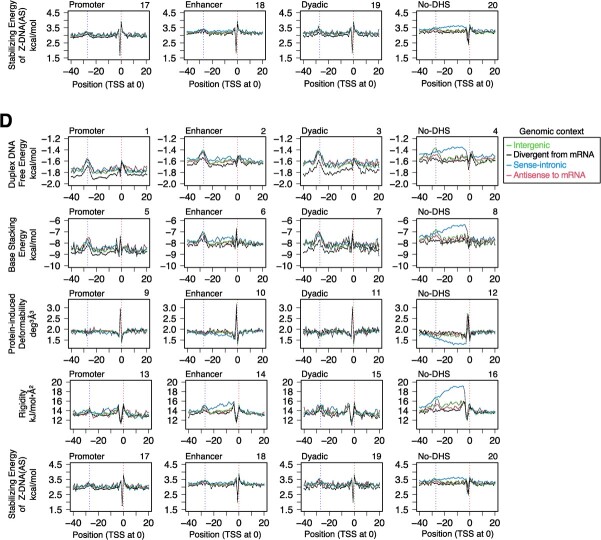
**CPEs and DPPs of human lncRNA gene promoters.** (**A**) The numbers of lncRNA genes in 16 categorized groups. The data of Extended Fig. 1c of Hon *et al*. *(**25**)* were modified. Definitions of the genomic context and the DHS type by Hon *et al*. *(**25**)* are as follows. ‘Divergent from mRNA’ (‘divergent lncRNAs’ in their study): the lncRNA genes occurring on the opposite strand of any mRNA genes or pseudogenes. ‘Sense-intronic lncRNA’: representatively, the lncRNA genes starting within the intron of another gene or those with at least 1/2 of their genic region overlapping with the genic region of any another gene. For the other species, see Hon *et al*. *(**25**)*. ‘Antisense to mRNA’ (‘antisense lncRNAs’ in their study): ≥50% genic regions of the lncRNA genes overlapping with those of mRNA genes or pseudogenes on the opposite strand. ‘Intergenic’ (‘intergenic lncRNAs’ in their study): the lncRNA genes on the outside of the above categories. The DHS types of lncRNA genes depend on where the DNase I hypersensitive sites (DHSs) occur and were classified as promoter, enhancer and 'dyadic' *(**25**)*. Among them, the term 'dyadic' is used when the category can be both promoter and enhancer. (**B**) Classification of promoters by CPEs. The classification was performed group by group. The term ‘core-less’ refers to the promoters that lack CPEs. Only the percentages of the top five promoter ‘species’ are shown. (**C, D**) Average DPP profiles: **C**, profiles of 27,919 unsorted promoters; **D**, profiles of 15,820 core-less promoters. The five DPPs were duplex DNA free energy *(**28**)*, base stacking energy *(**29**)*, protein-induced deformability *(**30**)*, rigidity *(**31**)*, and stabilizing energy of Z-DNA (AS) *(**32**)*. Promoters were aligned with the TSSs assigned at 0. Only mean values are shown. Two vertical dotted lines indicate positions 0 and −27, respectively. For the profiles of a wider DNA range and the data for SD, see Supplementary Figs. S1A, B and Supplementary Materials S4A-J, respectively.

### Average DPP profiles of promoters

Using the data sets of the DPPs, given as di- or tetra-nucleotide step values, each promoter sequence was converted into a chain of numerical values for each DPP (frameshift, 1 bp). After collecting data for a given set of promoters, the mean value and standard deviation at each position were calculated.

## Results

### Profiles of CPEs and DPPs of human lncRNA gene promoters

Cap Analysis of Gene Expression (CAGE) is regarded as the best method to identify the 5′-ends of mRNAs *(**33**)*. Since the genes encoding lncRNAs and miRNAs are transcribed by Pol II and processed in the same way as mRNA genes, we used the FANTOM CAT robust cut-off data reported by Hon *et al. (**25**)* to determine the promoter positions of human lncRNA genes. In this study, lncRNAs were classified into 16 groups, based on the combination of four genomic contexts and four DHS types (Fig. 1A). First, the promoters of lncRNA genes in the respective groups were sorted according to the CPE species, allowing a position deviation of ±5 bp from their canonical positions, which generated the 10–40 promoter types (Supplementary Material S1). Among the 16 groups, the resulting data were similar. In most groups, the promoters that lacked CPEs, named core-less promoters according to the naming for the corresponding promoters of mRNA genes *(**20**)*, accounted for more than 50% (Fig. 1B). The second most abundant promoters were usually single Inr-containing promoters, which accounted for ~20–~30% in most cases. Single BREd (TFIIB recognition element, downstream)-containing promoters or single DPE-containing promoters followed with much smaller percentages.

**Fig. 2:**
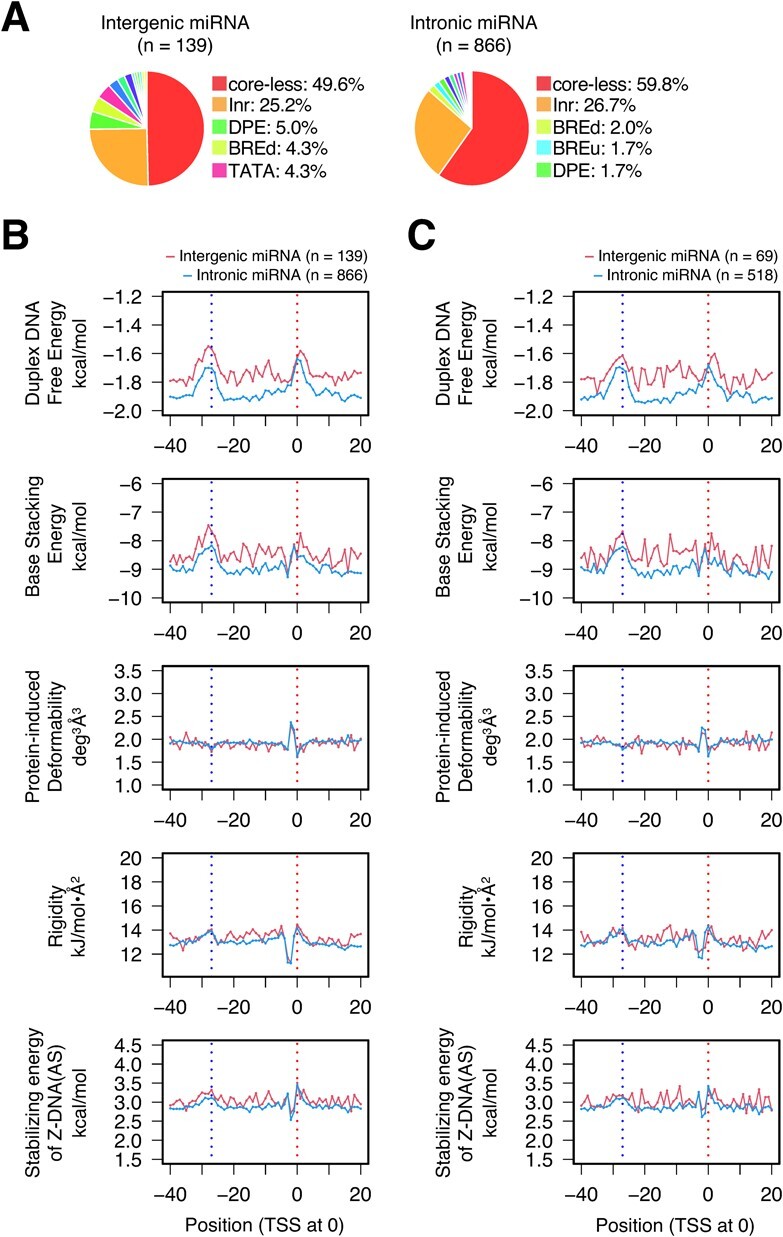
**CPEs and DPPs of human miRNA gene promoters.** (**A**) Classification of promoters by CPEs. The human miRNA genes were divided into two groups: intergenic and intronic *(**16**)*. The classification was performed group by group. Only the percentages of the top five promoter species are shown. For all data, see Supplementary Material S2. (**B, C**) Average DPP profiles: **B**, profiles of 1005 unsorted promoters; **C**, profiles of 587 core-less promoters. Promoters were aligned with the TSSs assigned at 0. Only mean values are shown. Two vertical dotted lines indicate positions 0 and −27, respectively. For the profiles of a wider DNA range and the data for SD, see Supplementary Figs. S2A, B and Supplementary Materials S5A-J, respectively.

**Fig. 3:**
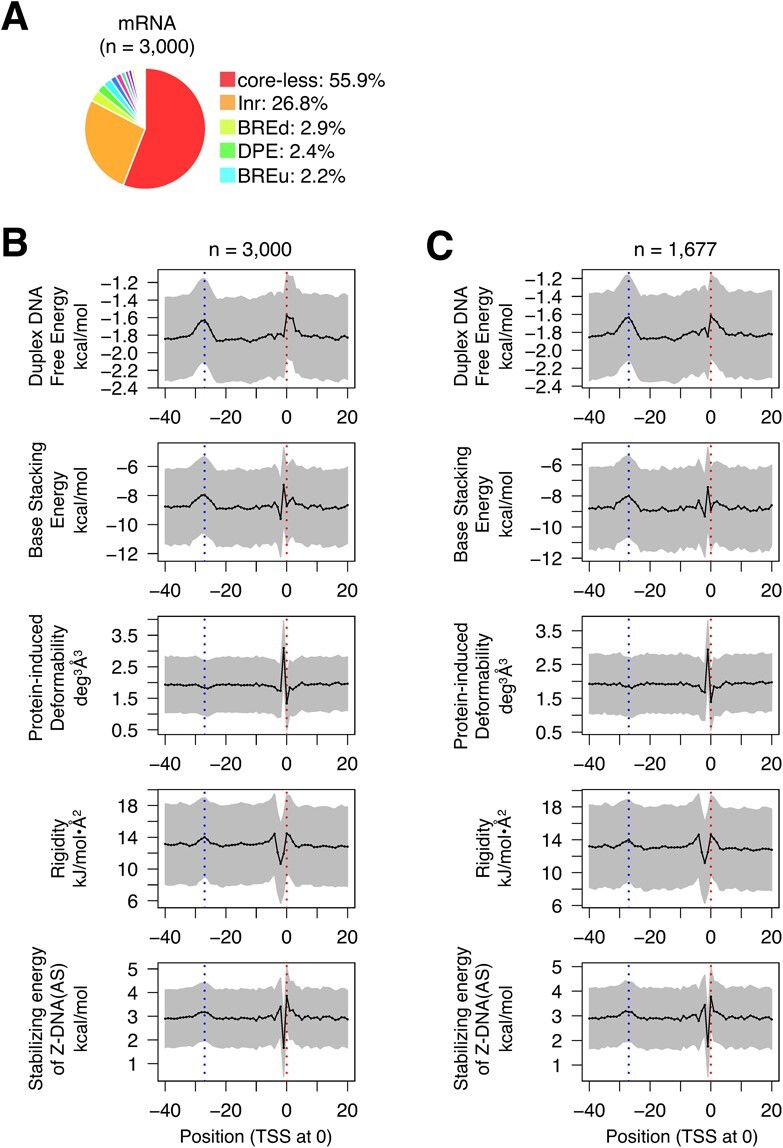
**CPEs and DPPs of human mRNA gene promoters.** (**A**) Classification of promoters by CPEs. Randomly selected promoters (*n* = 3000) were subjected to the analysis. Only the percentages of the top five promoter species are shown. For all data, see Supplementary Material S3. (**B, C**) Average DPP profiles: **B**, profiles of the 3000 unsorted promoters; **C**, profiles of 1677 core-less promoters. Promoters were aligned with the TSSs assigned at 0. Means ± SD are shown. Two vertical dotted lines indicate positions 0 and −27, respectively. For the profiles of a wider DNA range, see Supplementary Figs. S3A, B.

Subsequently, a set of unsorted promoters and that of core-less promoters in each group were subjected to average DPP profile calculations using five parameters: duplex DNA free energy, base stacking energy, protein-induced deformability, rigidity, and stabilizing energy of Z-DNA (AS); each set generated 80 profiles (Figs. 1C, D; for the wider range data, Supplementary Figs. S1A, B; for S.D., Supplementary Materials S4A-J). The resulting profiles were compared in DPP parameter-focused, genomic context-focused, and DHS type-focused manners. The data for a set of unsorted promoters are shown in Fig. 1C, Supplementary Fig. S1A, and Supplementary Materials S4A–E). In these parameter-focused comparisons, regardless of the different parameters, a marked peak or cleft was found around the TSS, and an additional low peak or hollow was also present around position −27, though it was small in the protein-induced deformability and stabilizing energy of Z-DNA (AS) profiles. The peak/cleft around the TSS was very sharp, except for the duplex DNA free energy profiles. The panel-by-panel genomic context-based comparison (comparison of data curve profiles) clarified the followings. In panels 1, 2, 3, 5 and 7, the baselines of the genomic context ‘divergent from mRNA’ were lower than those of the other genomic contexts, and in panels 4, 6, 8, 12, 14, 16 and 20, the genomic context ‘sense-intronic’ formed a ‘tableland’ or hollow in the region spanning from positions ~ 0 (TSS) to ~−30 (or more upstream in 4 and 8), usually for ‘no DHS’ of DHS type (panels 4, 8, 12, 16 and 20) and sometimes for ‘enhancer’ (panels 6 and 14). Lastly, the comparison based on DHS type showed that the duplex DNA free energy and base stacking energy of ‘promoter’ were lower than those of the other regions (compare panel 1 with panels 2, 3 and 4, and panel 5 with panels 6, 7 and 8). The corresponding 80 data plots of core-less promoters were indistinguishable from those of the unsorted promoters described above (Fig. 1D, Supplementary Fig. S1B, and Supplementary Materials S4F-J).

**Fig. 4:**
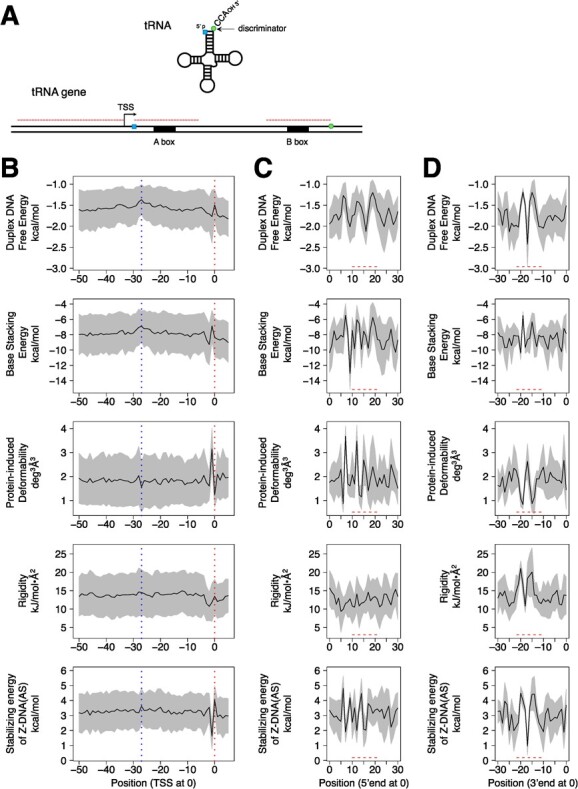
**Average DPP profiles of 5′-flanking and internal promoter regions of human tRNA genes. (A)** Diagram illustrating the three regions used for the acquisition of DPP profiles. **(B)** Profiles of the region spanning from the TSS to position −50 relative to the TSS (defined as 0). The number of samples was 445. Means ± SD are shown. Two vertical dotted lines indicate positions 0 and −27, respectively. **(C)** Profiles of the A-box-containing region spanning from the position corresponding to the 5′-end of the tRNA (defined as 0) to +30, relative to position 0. The A-box region is indicated with a dashed line. **(D)** Profiles of the B-box-containing region spanning from the position corresponding to the discriminator base of the tRNA (defined as 0) to −30, relative to position 0. The B-box region is indicated with a dashed line.

### CPE and DPP profiles of miRNA and mRNA gene promoters are similar to those of lncRNA gene promoters

MiRNAs are divided into ‘intergenic’ and ‘intronic’ *(**16**)*. CPE-based sorting of their gene promoters also showed that in each group, the core-less promoters and Inr-containing promoters included about one-half or more and a quarter of all promoters, respectively (Fig. 2A), similar to the data for lncRNA gene promoters (Fig. 1B). Although the small numbers of samples resulted in noisy plot curves, the distinctive characteristics of the DPP profiles, as described for the lncRNA gene promoters above, were obviously conserved in the promoters of both intergenic and intronic miRNA genes, irrespective of unsorted promoters and core-less promoters (Figs. 2B, C; Supplementary Figs. S2A, B; Supplementary Materials S5A–J). We also calculated the DPP profiles of 3000 unsorted and 1677 core-less human mRNA gene promoters, using randomly collected samples. Interestingly, in terms of the ratios of promoter species and DPP profiles, the resulting data were almost the same as the corresponding data for the promoters of lncRNA and miRNA genes (Fig. 3; Supplementary Fig. S3). Taken together, the promoters of lncRNA, miRNA and mRNA genes generally share the same DPP background. In other words, the core promoters of mRNA genes do not seem to be specialized to transcribe mRNAs.

### 5′-flanking regions of tRNA genes also have two distinctive sites in DPP profiles

RNA polymerase III (Pol III) also generates non-coding RNAs. The Pol III promoters are classified into three types: 5S rRNA gene type, tRNA gene type and U6 snRNA gene type. Among them, we focused on tRNA genes and subjected them to the same DPP analyses. The CPEs of tRNA gene promoters, called the A-box and B-box *(**34*–*39**)*, are located within their bodies (*i.e.*, gene-internal promoter). After transcription, the A-box forms the upstream part of the D-stem and part of the D-loop, and the B-box forms the whole TΨC-loop and part of the TΨC-stem *(**35**)*. Furthermore, the D-loop portion in the former contains the conserved GG sequence that is indispensable to form the L-shaped 3D structure *(**40**,**41**)*. The spacing between the A- and B-boxes is quite irregular, because a certain population of genes has an intron within the region and the size of the variable-loop is not fixed. Thus, for the tRNA genes, the calculations of DPP profiles targeted three regions: from position −50 to the TSS, the A-box-containing 30 bp region, and the B-box-containing 30 bp region (Fig. 4A). To obtain information from the average DPP profiles, the alignment of samples at a definite position is indispensable. Thus, we adopted the following positions for the points of alignment (*i.e.*, position 0): 1, the TSS; 2, the position corresponding to the 5′-end of tRNA; and 3, the position corresponding to the discriminator base of tRNA. The first group (upstream of the TSS) provided the data corresponding to the core promoters of Pol II genes. Interestingly, in these DPP profiles, peculiarity around the TSS were also very clear (Fig. 4B). We could also find ‘traces’ of low peak or hollow around position −27 in the DPP profiles. Interestingly, they corresponded to the shapes of low peak or hollow for the promoters of lncRNA, miRNA and mRNA genes. The second region contains the A-box with the consensus sequence ‘TGGCNNAGTGG’ *(**36**,**42**)* or ‘TRGYNNARBGG’ *(**43**)* (N = A or G or C or T; R = A or G; Y = C or T; B = C or G or T), and the third region has the B-box with the consensus sequence ‘GGTTCGANNCC’ *(**36**,**42**)*. The DPP profiles of these regions merely showed the properties of A- and B-box sequences. However, the end regions of these consensus sequences seemed highly unstable, as the end region of each box had a peak in the duplex DNA free energy and base stacking energy profiles.

Finally, we calculated sequence logos (Supplementary Fig. S4), which revealed a very slight nucleotide preference around the TSS: lncRNA genes, A or G at the TSS and C or T at −1; miRNA genes, C at −2; mRNA genes, A or G at the TSS and C or T at −1; tRNA genes, A or G at the TSS and C or T at −1. We hypothesize that these nucleotides are positively employed in the relevant promoters to generate the specific DPPs around the TSSs described above.

## Discussion

The current study showed for the first time that the promoters of lncRNA, miRNA and mRNA genes have two common distinctive regions in their DPP profiles: around the TSS and position −27. Furthermore, there was no noteworthy difference in the occurrence of CPEs in the promoters of these three classes of genes: core-less promoters were most prevalent (more than 50% in total), Inr-containing promoters were the second most abundant (~20—~30%), and the percentages of the other CPE(s)-containing promoters were much smaller and similar to one another over the classes (Figs. 1B, 2A, 3A). Thus, there are apparently no substantial differences in both the CPE and DPP profiles of the promoters in these three classes of genes, strongly indicating that the fundamental prerequisites for the function of promoter DNA are the same among them. Interestingly, tRNA genes also have distinctive DPPs around both positions, but to a lesser extent around −27. As a further note, in Fig. 1A, the lncRNA promoters of the DHS type ‘promoter’ *(**25**)* showed slightly lower base lines in the data curves of duplex DNA free energy and base stacking energy (Figs. 1C, D), which seem to suggest that the lncRNA promoters of this type are energetically more stable than the other promoters. The same suggestion is also possible for the genomic context ‘divergent from mRNA’, when compared to the other three genomic contexts. The current study also clarified that promoters with no DHS usually have unusual DPPs in the region from 0 to −30, in addition to those around the TSS and −27 (Figs. 1C, D). They may use these unusual DPPs to facilitate transcription initiation, instead of forming a DHS, by an unknown mechanism.

In each class of genes, unsorted promoters showed indistinguishable DPP profiles to those of core-less promoters, meaning that Inr sequences were ‘neutral’ to the DPP profiles around the TSS. Indeed, Inr-containing promoters also had almost the same DPP profiles (data not shown), suggesting that the DPPs themselves around the TSS seem to be generally more important in transcription than the Inr sequences themselves. The same seems true and clearer for the TATA box: the populations of TATA-box-containing promoters were very small (Supplementary Materials S1–S3), but the corresponding region (around −27) had a clear DPP ‘mark’ in most cases. The TATA-binding protein (TBP), a subunit of TFIID, severely bent several bps centered around −27 in the PIC, irrespective of the presence of the TATA box *(**44**)*. Furthermore, a recent study suggested that the DNA distortion induced by PIC may lead to strand separation around the TSS; *i.e.*, promoter opening *(**45**)*. Taken together, the distinctive DPPs around the TSS and −27 are hypothesized to be prerequisites to ease DNA bending and melting in Pol II transcription, respectively. These properties may also affect chromatin architecture itself of the core promoter region before transcription.

The upstream regions of tRNA genes also had distinctive DPPs around the TSS and −27, although the latter was not as prominent as compared to those of the Pol II promoters of the three classes. To initiate tRNA gene transcription, Pol III requires TFIIIB, a protein complex containing TBP, which recruits Pol III and promotes open complex formation *(**46**)*. The current study showed that the TSSs of tRNA genes have the same DPPs as those of the Pol II genes. Almost the same seems true for the TBP binding sites of tRNA genes. Thus, we hypothesize that the distinctive DPPs also function to facilitate DNA bending near −27 and strand separation around the TSS over the kingdoms of Pol II and Pol III. This study also revealed that the DPP profiles of A- and B-boxes of tRNA suggested that they are highly unstable in the DNA duplex (Figs. 4C, D). To ease strand separation, the DPPs of A- and B-boxes may also be used in the Pol III transcription system.

## Supplementary Data

Supplementary Data are available at *JB* Online.

## Funding

This work was supported by JSPS KAKENHI (grant no. 19H04391 to T.O.) and JST SPRING (grant no. JPMJSP2128 to K.U.).

## Conflict of Interest

The authors declare no conflict of interest.

## Authors' Contributions

T.O. and K.U. conceived and designed the study. K.U. and T.O. analyzed the data and outputted the figures and tables. K.U. and T.O. wrote the manuscript. T.O. supervised the study and polished the paper.

## Data availability

Supplementary materials (Supplementary materials.xlsx) for Figs. S1, S2 and S3A are available at http://doi.org/10.6084/m9.figshare.24452788.

## Supplementary Material

Web_Material_mvad111
