# Supplementary material for: Distinctive physical properties of DNA shared by RNA polymerase II gene promoters and 5′-flanking regions of tRNA genes
Source: J Biochem. 2023 Dec 15;175(4):395–404. doi: 10.1093/jb/mvad111 (PMC11005993; doi:10.1093/jb/mvad111)
Supplement: Web_Material_mvad111 [file web_material_mvad111.pdf]

Supplementary figures

Distinctive physical properties of DNA shared by RNA polymerase II gene promoters and 5´-flanking regions of tRNA genes

Kohei Uemura[1], **Takashi Ohyama**[1,2,*]

[1] Major in Integrative Bioscience and Biomedical Engineering, Graduate School of Science and Engineering, Waseda University, 2-2 Wakamatsu-cho, Shinjuku-ku, Tokyo 162-8480, Japan

[2] Department of Biology, Faculty of Education and Integrated Arts and Sciences, Waseda University, 2-2 Wakamatsu-cho, Shinjuku-ku, Tokyo 162-8480, Japan

* To whom correspondence should be addressed. E-mail: ohyama@waseda.jp

Suppl. Fig.1

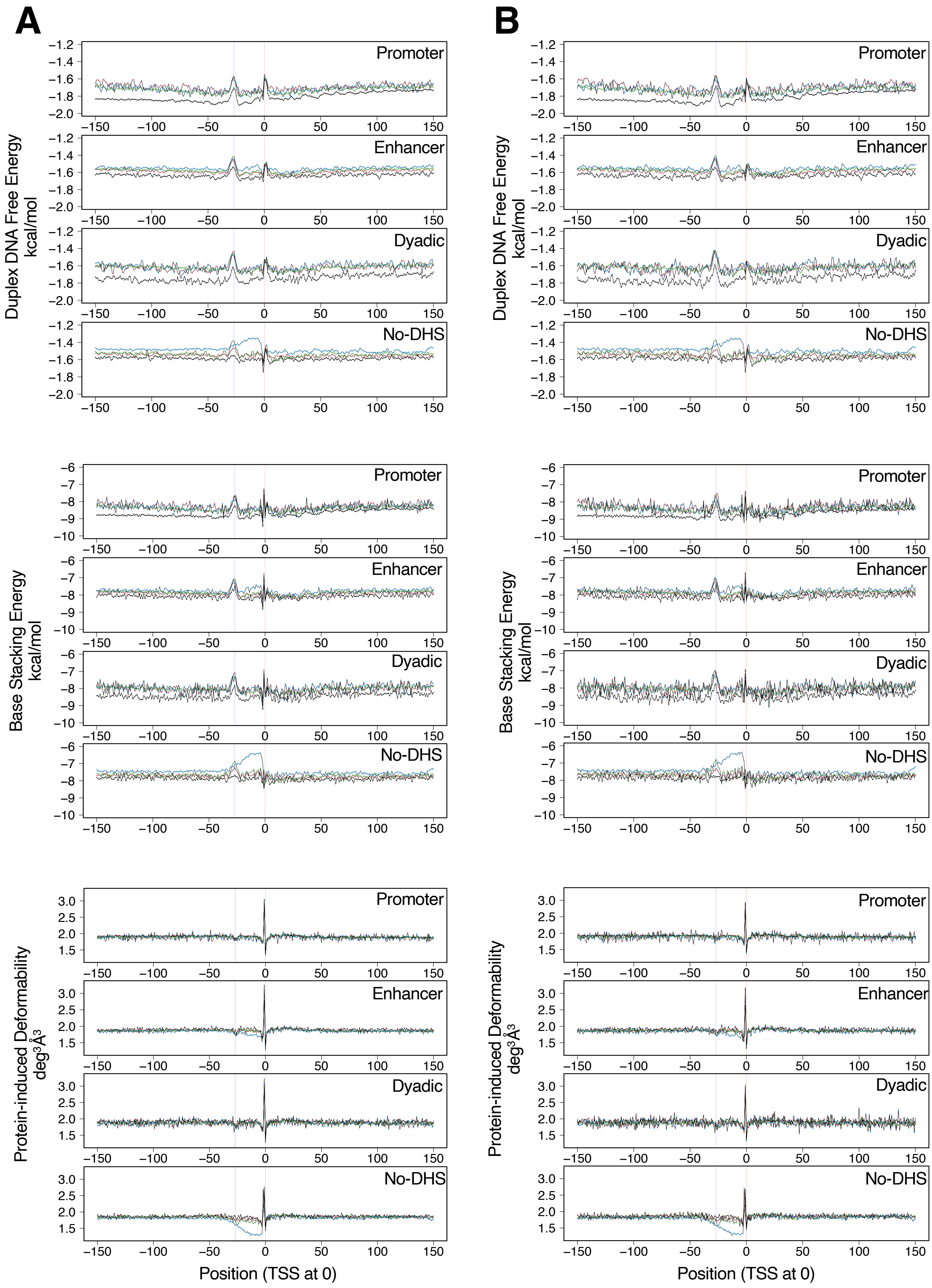

A
B
Promoter
Enhancer
Dyadic
No-DHS
Duplex DNA Free Energy kcal/mol
Base Stacking Energy kcal/mol
Protein-induced Deformability deg³Å³
Position (TSS at 0)

Suppl. Fig.1 continued

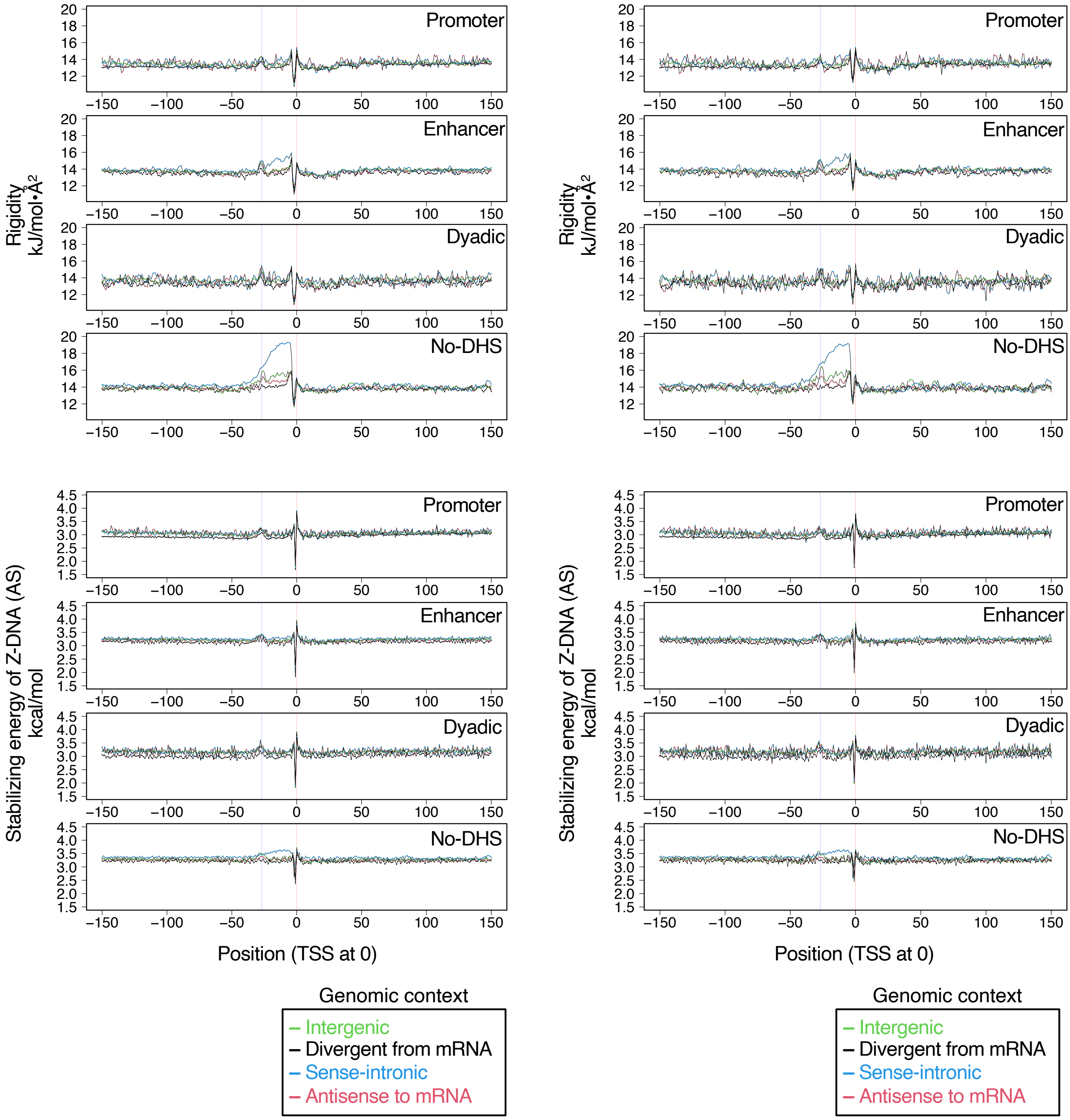

Supplementary Figure 1. **Average DPP profiles of the region from –150 to + 150 of human lncRNA genes** **(A)** Profiles of unsorted promoters of 27,919. **(B)** Profiles of core-less promoters of 15,820. Five DPPs were duplex DNA free energy, base stacking energy, protein-induced deformability, rigidity, stabilizing energy of Z-DNA (AS). Genes were aligned with the TSSs assigned at 0. Only mean values are shown. Red and blue dotted lines indicate positions 0 and –27, respectively.

Suppl. Fig. 2

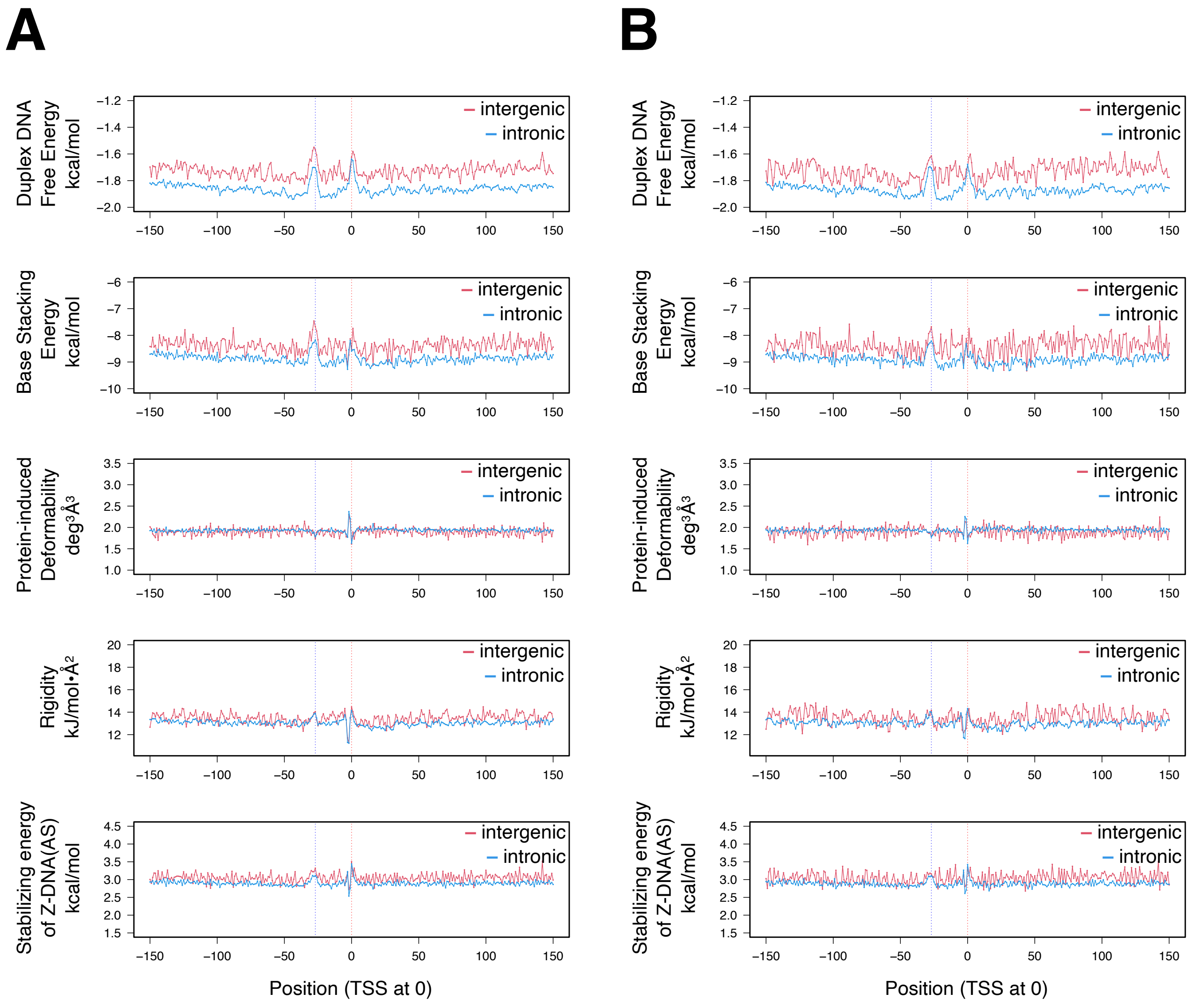

Supplementary Figure 2. Average DPP profiles of the region from –150 to + 150 of human miRNA genes
(A) Profiles of unsorted promoters of 1,005. (**B)** Profiles of core-less promoters of 587. Genes were aligned with the TSSs assigned at 0. Only mean values are shown. Red and blue dotted lines indicate positions 0 and –27, respectively.

Suppl. Fig. 3

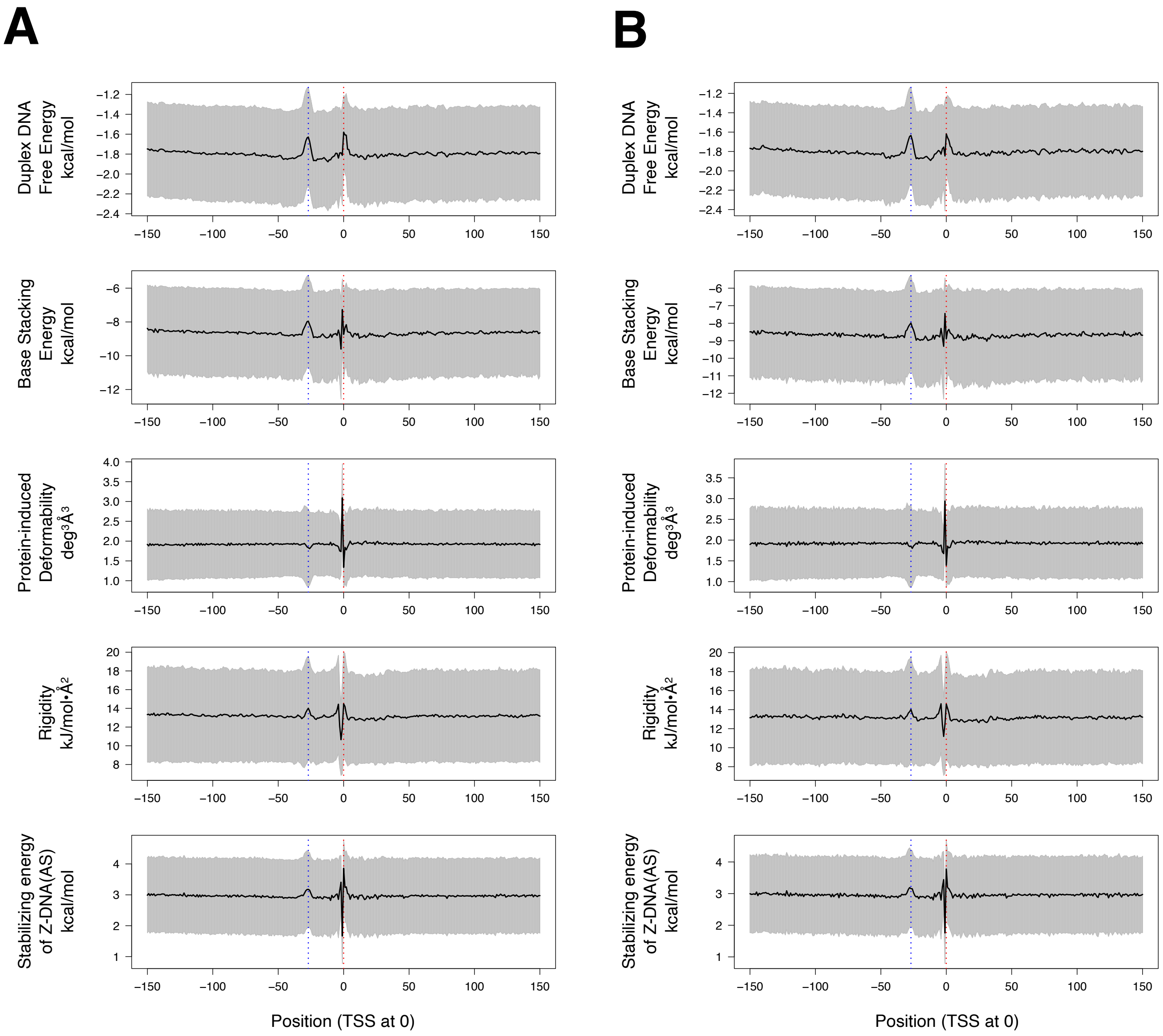

Supplementary Figure 3. Average DPP profiles of the region from –150 to + 150 of human mRNA genes

(A) Profiles of unsorted promoters of 3,000. **(B)** Profiles of core-less promoters of 1,677. Genes were aligned with the TSSs assigned at 0. Means ± SD are shown. Red and blue dotted lines indicate positions 0 and –27, respectively.

Suppl. Fig. 4

A

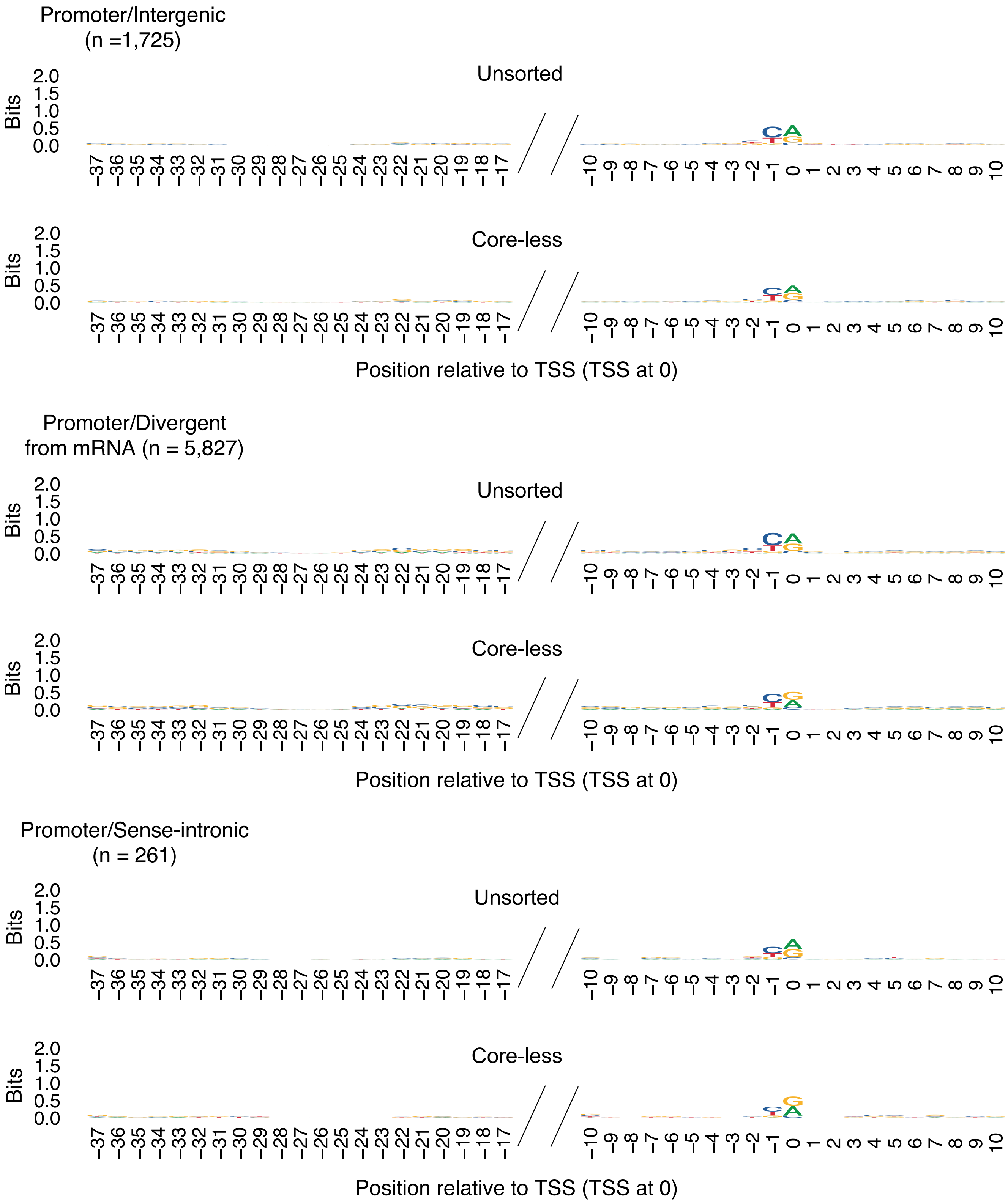

Promoter/Intergenic
(n =1,725)
Unsorted
Core-less
Bits
2.0
1.5
1.0
0.5
0.0
Position relative to TSS (TSS at 0)
Promoter/Divergent
from mRNA (n = 5,827)
Unsorted
Core-less
Position relative to TSS (TSS at 0)
Promoter/Sense-intronic
(n = 261)
Unsorted
Core-less
Position relative to TSS (TSS at 0)

Suppl. Fig. 4 continued

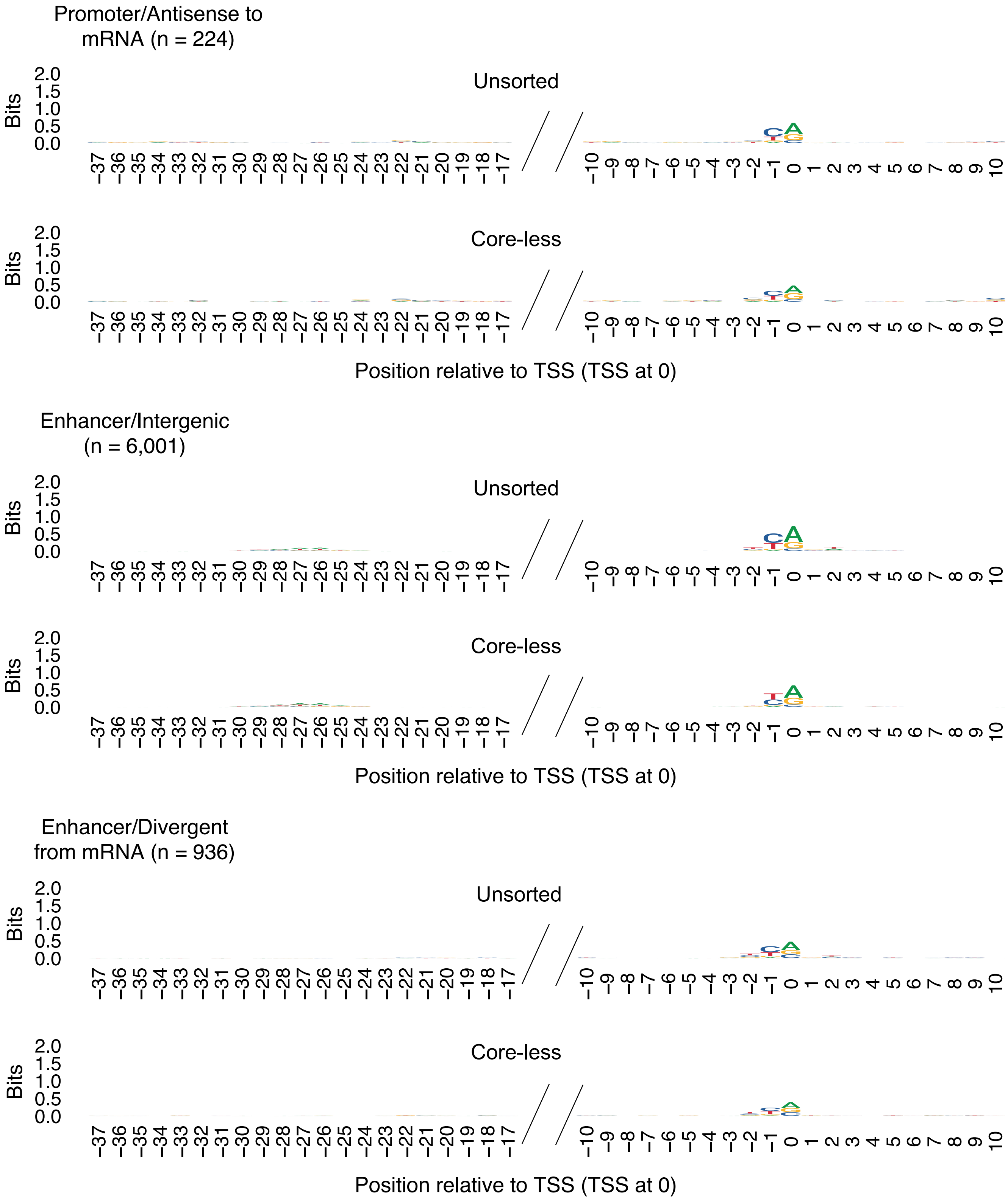

Suppl. Fig. 4 continued

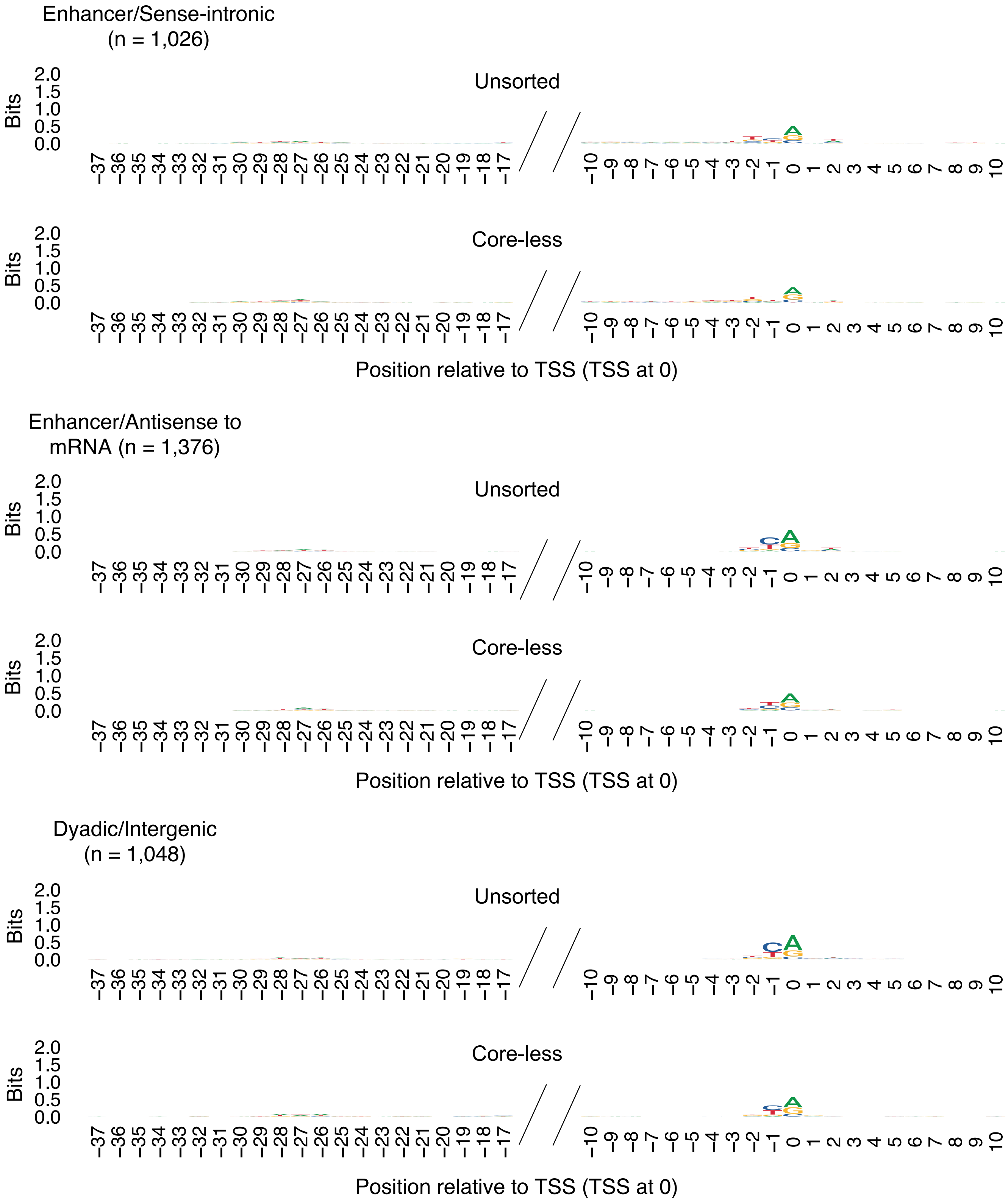

Suppl. Fig. 4 continued

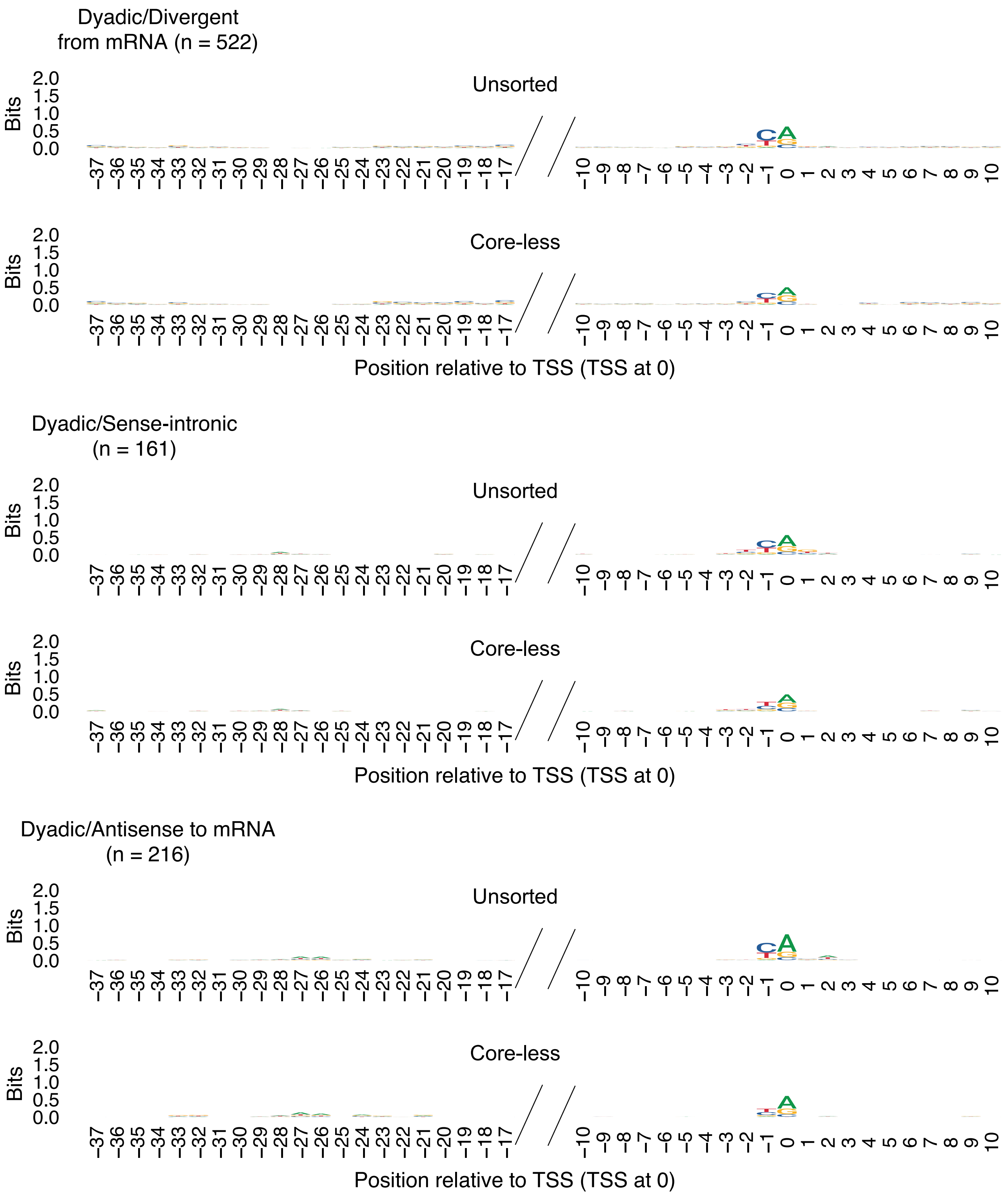

Suppl. Fig. 4 continued

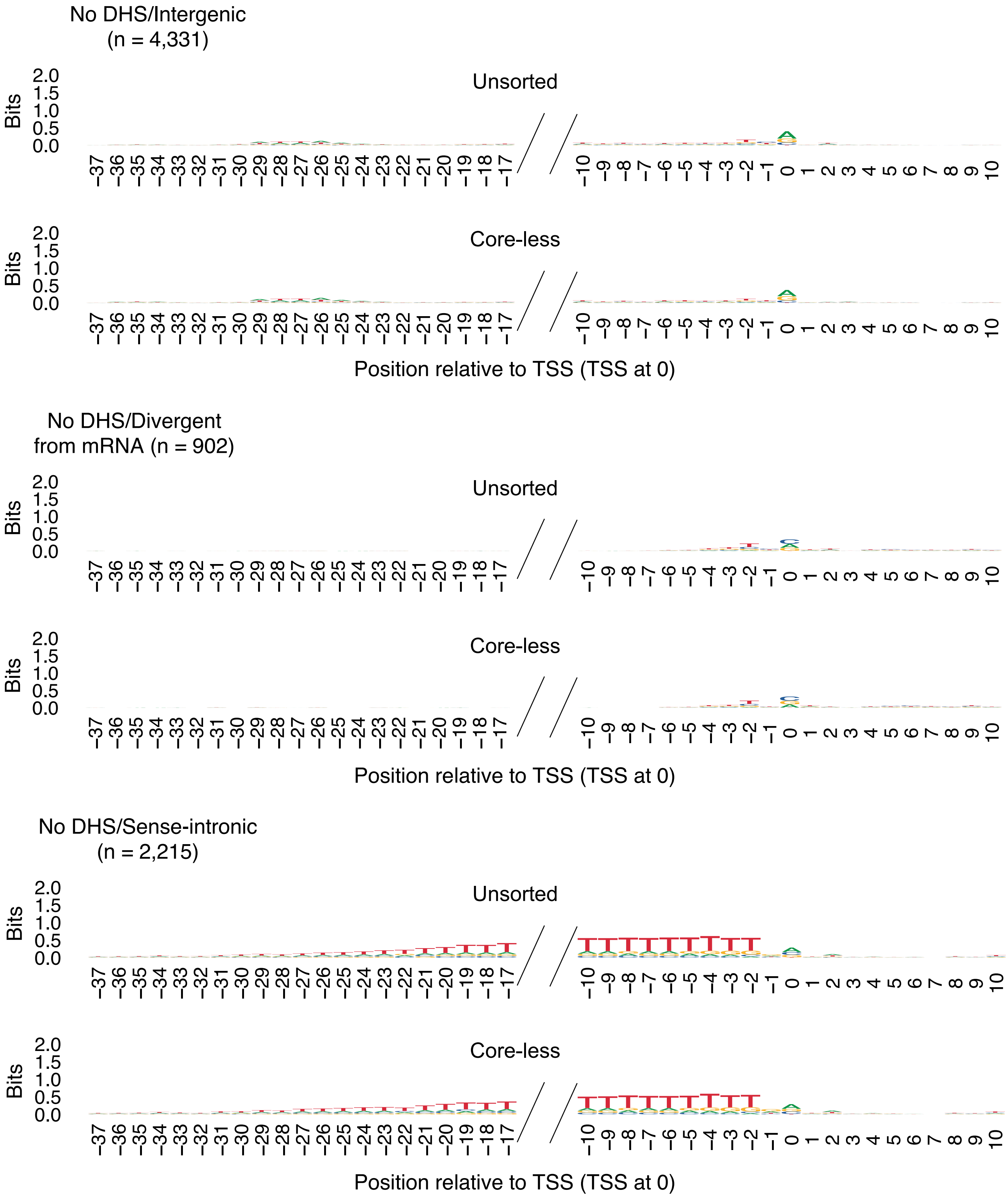

Suppl. Fig. 4 continued

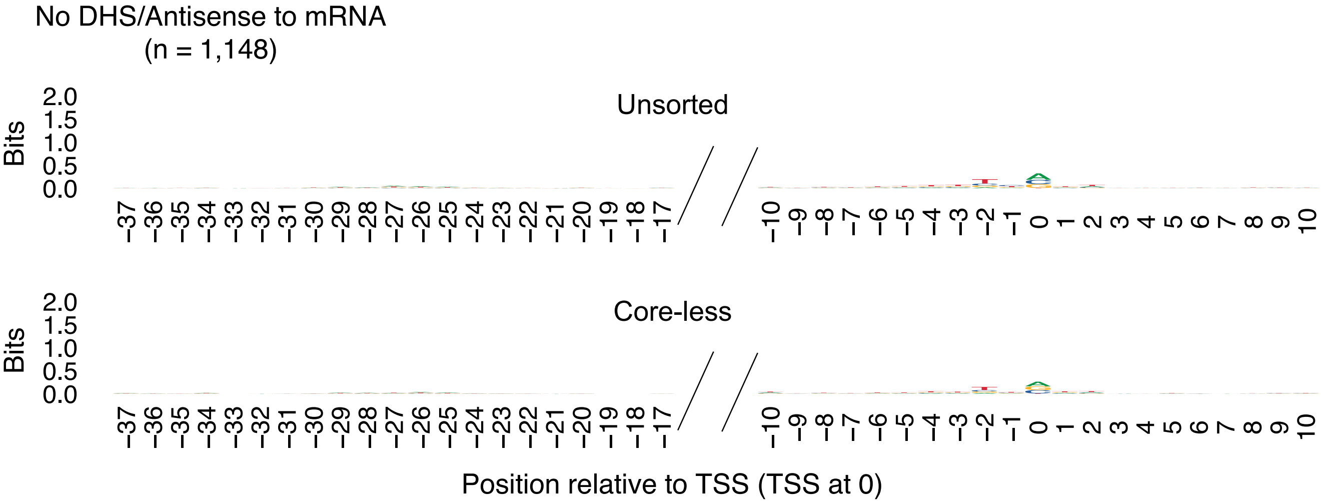

B

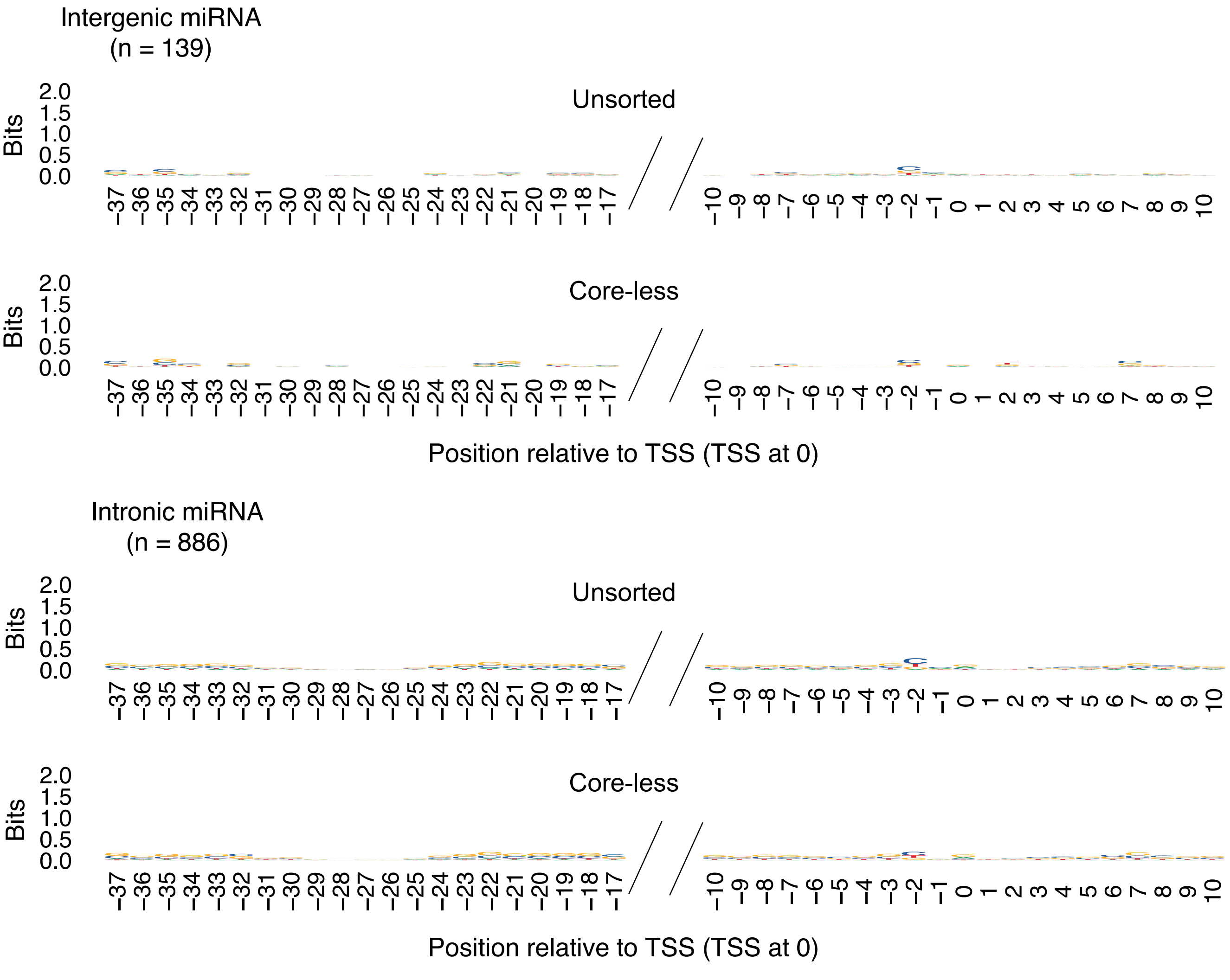

Suppl. Fig. 4 continued

C

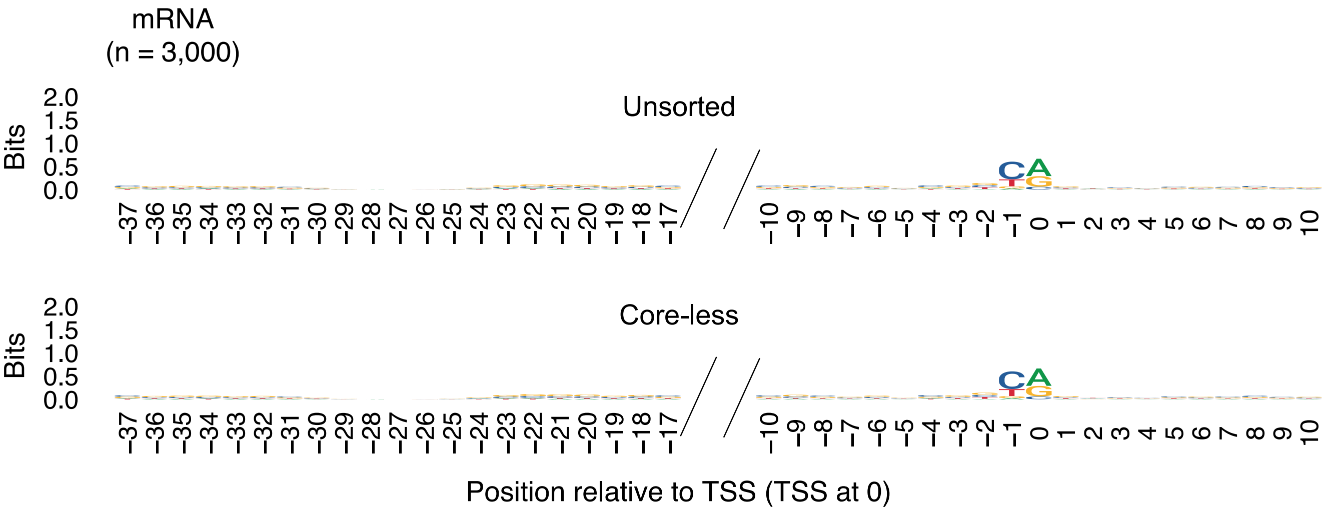

D

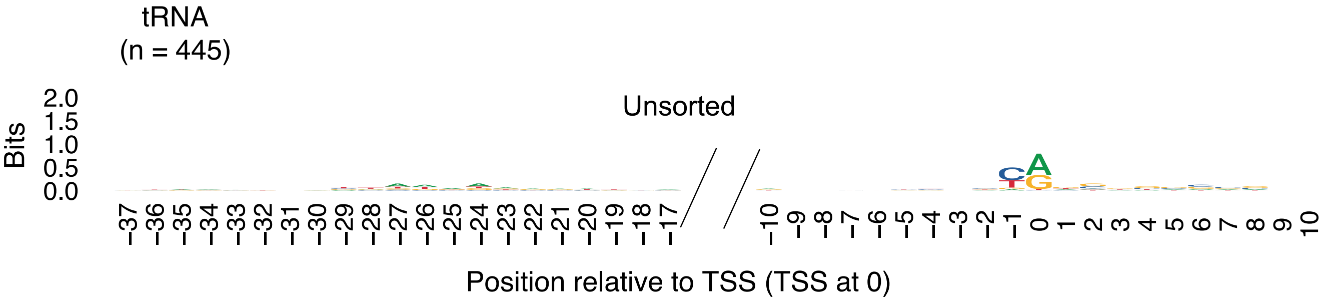

Supplementary Figure 4. Sequence logos of the regions around the TSS and –27 for human lncRNA (A), miRNA (B), mRNA (C) and tRNA genes (D).

Genes were aligned with the TSSs assigned at 0. Sequence logos were generated using the R package “ggseqlogo”.
